# Isobutyric acid enhances the anti-tumour effect of anti-PD-1 antibody

**DOI:** 10.1038/s41598-024-59677-1

**Published:** 2024-05-17

**Authors:** Masakazu Murayama, Masahiro Hosonuma, Atsuo Kuramasu, Sei Kobayashi, Akiko Sasaki, Yuta Baba, Yoichiro Narikawa, Hitoshi Toyoda, Junya Isobe, Eiji Funayama, Kohei Tajima, Aya Sasaki, Yuki Maruyama, Yoshitaka Yamazaki, Midori Shida, Kazuyuki Hamada, Yuya Hirasawa, Toshiaki Tsurui, Hirotsugu Ariizumi, Tomoyuki Ishiguro, Risako Suzuki, Ryotaro Ohkuma, Yutaro Kubota, Atsushi Horiike, Takehiko Sambe, Mayumi Tsuji, Satoshi Wada, Shinichi Kobayashi, Toshikazu Shimane, Takuya Tsunoda, Hitome Kobayashi, Yuji Kiuchi, Kiyoshi Yoshimura

**Affiliations:** 1 Department of Clinical Immuno Oncology, Clinical Research Institute for Clinical Pharmacology and Therapeutics, Showa University, 6-11-11, Kitakarasuyama, Setagaya-ku, Tokyo, 157-8577 Japan; 2 Division of Medical Pharmacology, Department of Pharmacology, Showa University School of Medicine, Tokyo, Japan; 3 Pharmacological Research Center, Showa University, Tokyo, Japan; 4 Department of Otorhinolaryngology-Head and Neck Surgery, Showa University School of Medicine, Tokyo, Japan; 5 Division of Medical Oncology, Department of Medicine, Showa University School of Medicine, Tokyo, Japan; 6 Department of Otorhinolaryngology, Fujigaoka Hospital, Yokohama, Japan; 7 Department of Orthopaedic Surgery, School of Medicine, Showa University, Tokyo, Japan; 8 Department of Hospital Pharmaceutics, School of Pharmacy, Showa University, Tokyo, Japan; 9 Division of Toxicology, Department of Pharmacology, Toxicology and Therapeutics, Showa University School of Pharmacy, Tokyo, Japan; 10 Division of Clinical Pharmacology, Department of Pharmacology, Showa University School of Medicine, Tokyo, Japan; 11 Department of Clinical Diagnostic Oncology, Clinical Research Institute for Clinical Pharmacology and Therapeutics, Showa University, Tokyo, Japan; 12 Clinical Research Institute for Clinical Pharmacology and Therapeutics, Showa University, Tokyo, Japan; 13 Head and Neck Oncology Center, Showa University, Tokyo, Japan; 14 Division of Pharmacology, Department of Pharmacology, Toxicology and Therapeutics, School of Pharmacy, Showa University, Tokyo, Japan

**Keywords:** Cancer, Cancer microenvironment, Cancer therapy, Head and neck cancer, Tumour immunology

## Abstract

The low response rate of immune checkpoint inhibitors (ICIs) is a challenge. The efficacy of ICIs is influenced by the tumour microenvironment, which is controlled by the gut microbiota. In particular, intestinal bacteria and their metabolites, such as short chain fatty acids (SCFAs), are important regulators of cancer immunity; however, our knowledge on the effects of individual SCFAs remains limited. Here, we show that isobutyric acid has the strongest effect among SCFAs on both immune activity and tumour growth. In vitro, cancer cell numbers were suppressed by approximately 75% in humans and mice compared with those in controls. Oral administration of isobutyric acid to carcinoma-bearing mice enhanced the effect of anti-PD-1 immunotherapy, reducing tumour volume by approximately 80% and 60% compared with those in the control group and anti-PD-1 antibody alone group, respectively. Taken together, these findings may support the development of novel cancer therapies that can improve the response rate to ICIs.

## Introduction

Immune checkpoint inhibitors (ICIs) maintain the anti-tumour effects of T cells by inhibiting mechanisms that restrict T cell activation and effector function. They have been used in a variety of cancers, but their low efficacy is a challenge^1^. The efficacy of ICIs is known to be dependent on the tumour microenvironment^2^; thus, an approach targeting the tumour microenvironment is necessary to improve efficacy.

The gut microbiota is known to significantly influence the immune function of the host^3^. Because it also affects the immune cell composition of the tumour microenvironment, the gut microbiota largely affects the efficacy of ICIs^4–8^. It has been reported that dysbiosis caused by antibiotic use reduces ICI efficacy^9,10^. Furthermore, ICI-resistant patients acquired responsiveness to ICI after transplantation with faecal microbiota from ICI-responsive patients^11,12^.

Several mechanisms are known to underlie host immune function regulation by the gut microbiota, one of which is the regulation by metabolites produced by the gut bacteria. Intestinal bacteria break down undigested host products, such as dietary fibre, to produce short chain fatty acids (SCFAs), which significantly affect host immune function^13^. For example, acetic acid, propionic acid, and butyric acid activate regulatory T cells and enhance effector T cell function^14–16^. In addition, valeric acid augments the anti-tumour effect of cytotoxic T cells^17^ and suppresses cancer cell proliferation^18,19^.

While straight SCFAs, such as acetic acid, propionic acid, and butyric acid, are derived from dietary fibre (non-digestible carbohydrates), branched SCFAs, such as isobutyric acid and isovaleric acid, are produced by the fermentation of branched chain amino acids (valine, leucine, and isoleucine) derived from non-digestible proteins. Branched SCFAs are present in smaller amounts in the digestive tract than other SCFAs, and their functions are largely unknown^20^.

In this study, to investigate the effects of SCFAs on host immune function, we screened seven SCFAs using an in vitro co-culture system of cancer cells and T cells. Among them, isobutyric acid, a branched SCFA, selectively inhibited cancer cell growth and synergistically potentiated the anti-tumour effect of anti-PD-1 antibody. These promising results may aid the development of a new combination therapy for improving the efficacy of ICIs.

## Results

### Selective inhibition of cancer cell survival by isobutyric acid

**Figure 1:**
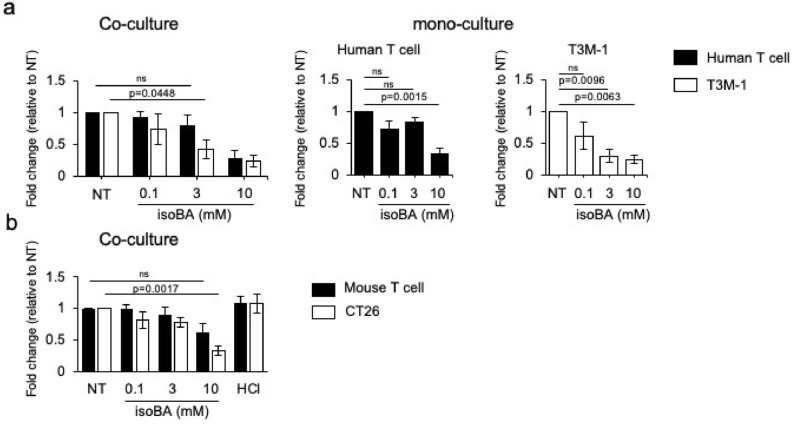
Preferential inhibition of cancer cell survival by isobutyric acid over that of T cells. (**a**) Human T cells (5 × 10^5^ cells) and T3M-1 Clone2 oral cancer cells (5 × 10^4^ cells) were co-cultured for 72 h with isobutyric acid at the indicated concentrations. The number of cancer and T cells was analysed by flow cytometry using fluorescent counting beads. CD45-positive cells were defined as T cells and all others as cancer cells. The graphs in the left, centre, and right represent cancer/T cell co-culture, T cell mono-culture, and cancer cell mono-culture, respectively. (**b**) Mouse T cells and CT-26 cancer cells were co-cultured for 72 h with isobutyric acid at the indicated concentrations. The number of cancer and T cells was analysed using flow cytometry. To examine the effect of acidic conditions, hydrochloric acid was added to induce a low pH as that induced by 10 mM isobutyric acid. Data are representative of three independent experiments. Error bars represent S.E.M. *NT* no treatment, *HCl* hydrochloric acid, *isoBA* isobutyric acid.

First, we examined the effects of seven SCFAs (acetic, propionic, butyric, isobutyric, valeric, isovaleric, and hexanoic acids) on the survival of T or cancer cells. T3M-1 is a cell line from squamous carcinoma of human head and neck cancer. T cells were derived from peripheral blood mononuclear cells (PBMCs) of healthy donors. All SCFAs tested in this study had a concentration-dependent inhibitory effects on the survival of T3M-1 cells after 3 days in mono-culture (Fig. 1a and Supplementary Fig. S1). Similarly, propionic, butyric, valeric, isovaleric, and hexanoic acids decreased the number of T cells in mono-culture in a concentration-dependent manner (Supplementary Fig. S1b–f). However, acetic and isobutyric acids had relatively small effect on the T cell number, even at the highest concentration (Fig. 1a and Supplementary Fig. S1a). Therefore, we chose these two SCFAs and further tested their effects on T cell numbers and cancer cells in a co-culture setting (Fig. 1a, Supplementary Figs. S1a, S2). Acetic acid selectively exerted an inhibitory effect on cancer cells only at 10 mM (Supplementary Fig. S1a). In contrast, isobutyric acid preferentially inhibited the survival of cancer cells at 3 and 10 mM (Fig. 1a).

Several studies have investigated the effects of acetic acid, but few have examined the effects of isobutyric acid in detail^13,16^. Therefore, we focused on isobutyric acid in the subsequent experiments. We next investigated the effect of isobutyric acid on the survival of the mouse colonic cancer cell line CT-26 and spleen-derived T cells in a co-culture setting. Both T cells and cancer cells were decreased after 3 days of co-culture in the presence of isobutyric acid in a concentration-dependent manner (Fig. 1b). However, isobutyric acid at 10 mM significantly decreased the number of CT-26 cells by more than 60%, whereas more than 60% of T cells survived in the same condition (Fig. 1b). It is of note that there was no growth inhibition when hydrochloric acid (HCl) was added to induce an acidic condition similar to that induced by 10 mM isobutyric acid, suggesting that the effect was due to isobutyric acid itself, and not the low pH. We further analysed the effect of isobutyric acid on additional human and mouse colon cancer cell lines in co-culture settings. Human colon cancer cell line SW480 survived at 3 mM isobutyric acid (Fig. S2b), contrasting the result obtained with T3M-1that was sensitive to isobutyric acid at the same concentration (Fig. 1a). This suggests some cancer cell lines are relatively resistant to isobutyric acid. Another mouse colon cancer cell line MC38 significantly decreased in number with 10 mM isobutyric acid (Fig. S2c), similar to the result with CT-26. Together with the results obtained with additional human and mouse cell lines, these results suggest that isobutyric acid preferentially inhibits the survival of cancer cells over that of T cells.

### Effect of isobutyric acid on the immune phenotype of T cells

**Figure 2:**
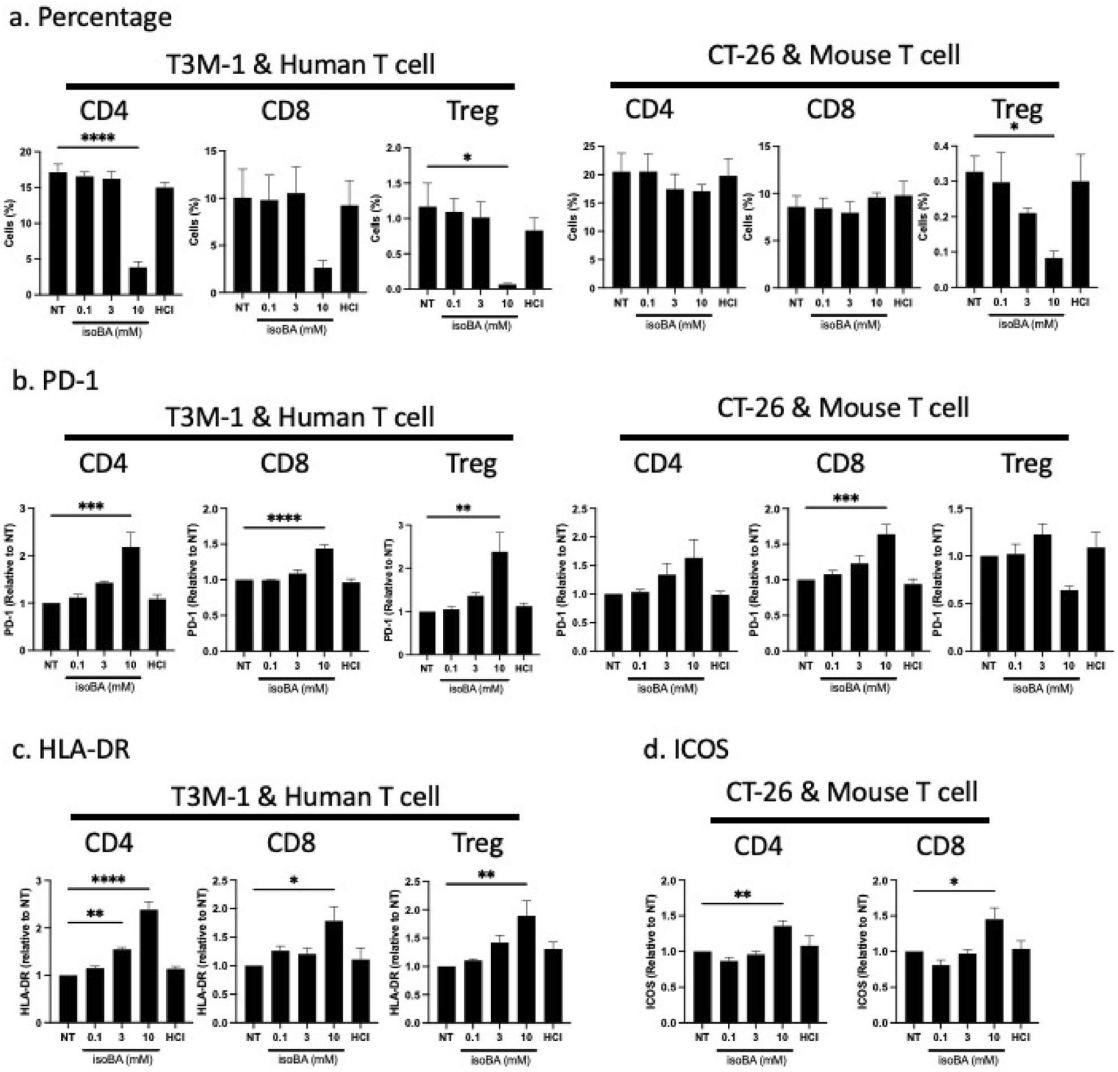
Characterisation of T cell populations upon treatment with isobutyric acid. Human or mouse T cells (5 × 10^5^ cells) were co-cultured with T3M-1 Clone2 oral cancer cells or CT-26 cells (5 × 10^4^ cells) respectively in the presence of different concentrations of isobutyric acid for 72 h. Afterwards, the T cell populations were evaluated based on the expression of surface markers using flow cytometry. (**a**) Percentage of CD4^+^ T cells, CD8^+^ T cells, and Treg cells present in the cancer/T cell co-cultures with isobutyric acid. (**b**–**d**) Expression of PD-1 (**b**) and HLA-DR (**c**) and ICOS (**d**) in CD4 + T cells, CD8 + T cells, and Treg cells. To examine the effect of acidic condition, hydrochloric acid was added to induce a low pH as that induced by 10 mM isobutyric acid. Data are mean of three independent experiments. Error bars represent S.E.M. *NT* no treatment, *HCl* 10 mM hydrochloric acid, *isoBA* isobutyric acid, *ns* not significant, *p < 0.05, **p < 0.01, ***p < 0.001, ****p < 0.0001 vs NT.

To characterize the phenotype of T cells under isobutyric acid treatment, we performed flow cytometric analysis of T cells in a co-culture setting. In human, both CD4 and CD8 T cells population were decreased at 10 mM isobutyric acid. However, the inhibitory effect of isobutyric acid on Treg (CD4 + CD25 + CD127- FoxP3 +) cells was more potent than on CD4 or CD8 T cells (Fig. 2a). This was confirmed by the experiment co-cultured with another cell line SW480 (Fig. S3b). In comparison to human T cells, mouse CD4 and CD8 T cells were relatively resistant, even at high concentrations of isobutyric acid (Fig. 2a). In contrast, isobutyric acid had concentration-dependent suppressive effects on mouse Treg cells (Fig. 2a). These result were partly reproduced by co-culture using another colon cancer cell line MC38 (Fig. S4b), suggesting that isobutyric acid had preferential inhibitory effect on Treg cells over other T cell populations.

Next, we analyzed expression of several activation markers on each T cell population. PD-1 expression was significantly increased by isobutyric acid in a concentration dependent manner in human T cells (Figs. 2b, S3c). In mouse, PD-1 expression in CD4 + and CD8 + T cells were increased upon co-culture with CT-26 in a concentration-dependent manner (Fig. 2b). However, the effect was not prominent in those cells co-cultured with MC38 cells (Fig. S4c). PD-1 expression in regulatory T cells were not increased in response to isobutyric acid and rather decreased when co-cultured with MC38 cells (Figs. 2b and S4c). Next, we measured HLA-DR as an activation marker for human T cell [Tippalagama et al. JI 2021]. Isobutyric acid significantly increased HLA-DR expression in human CD4 +, CD8 + T cells, and Treg cells concentration-dependently (Figs. 2c and S3d). Expression of another activation marker for T cell CD95 was not altered in human T cells by isobutyric acid except subtle decrease of CD8 + T cells at 10 mM when co-cultured with SW480 (Fig. S3e). However, in mouse T cells, isobutyric acid increased the expression of CD95 in some T cell populations (Fig. S4d). Further, inducible T cell co-stimulator (ICOS) was upregulated by 10 mM isobutyric acid treatment particularly both in CD4 + and CD8 + T cells in humans (Fig. 2d). The changes on these activation markers were not induced by hydrochloric acid treatment, suggesting that these effects were caused by isobutyric acid itself. Expressions of human CTLA-4, Ki67, and Tim3, as well as mouse CTLA-4, CD69, and ICOS, were not altered by the treatment with isobutyric acid (Fig. S5). Overall, these results suggest that isobutyric acid activates T cells in co-culture settings.

### Direct effect of isobutyric acid on the gene expression of T cells

**Figure 3:**
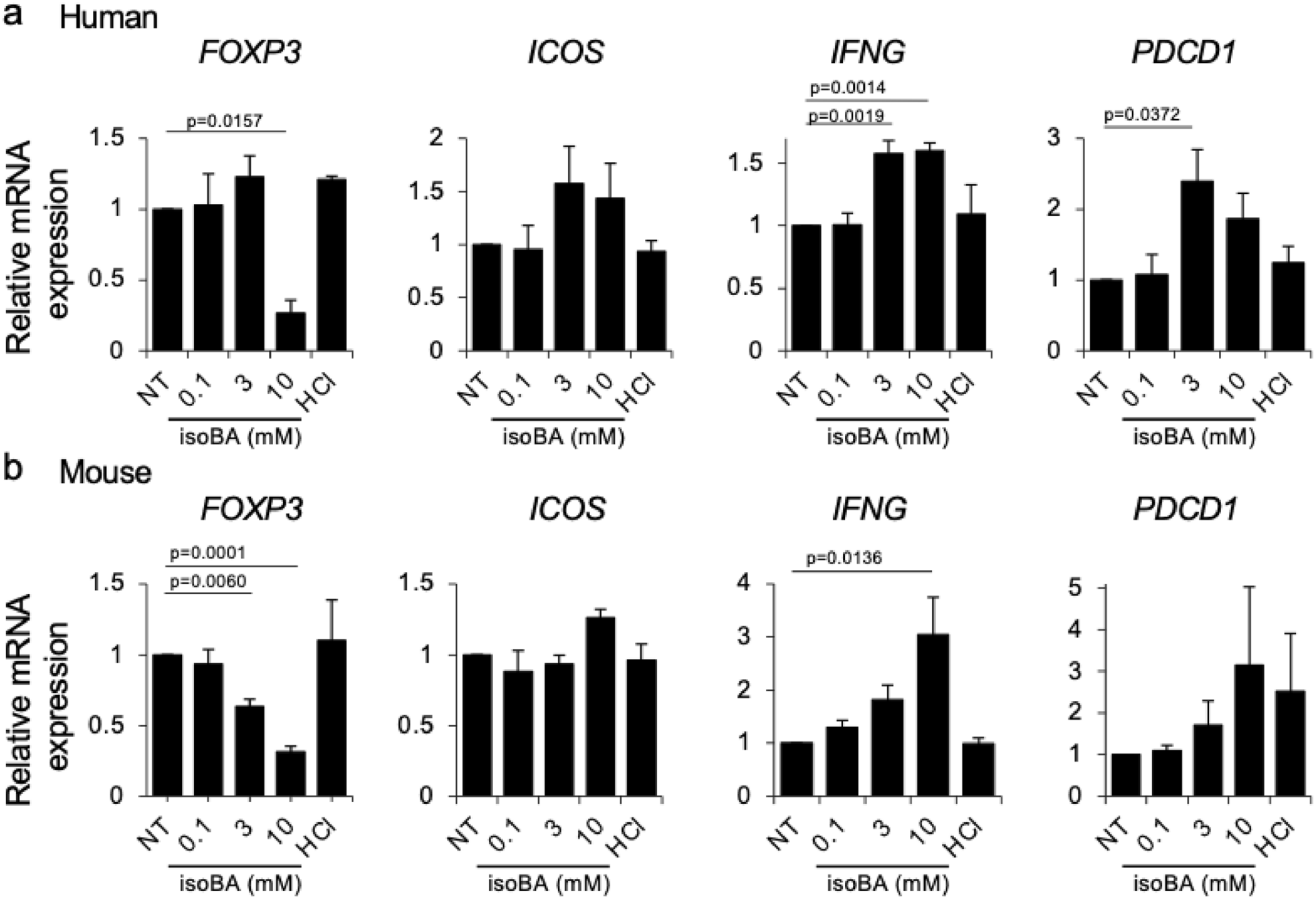
Direct effects of isobutyric acid on gene expression in T cells. Human and mouse T cells were cultured with various concentrations of isobutyric acid for 72 h before RNA extraction. Gene expression of *IFNG*, *ICOS*, *PDCD1*, and *FOXP3* in human (**a**) and mouse (**b**) T cells was measured using quantitative PCR. Data are presented as relative values against non-treated cells and are mean of three independent experiments. Error bars represent S.E.M. *NT* no treatment, *HCl* hydrochloric acid, *isoBA* isobutyric acid.

To investigate the mechanisms underlying changes in the T cell population in the co-culture setting, we treated mono-cultured T cells with various concentrations of isobutyric acid and quantified the mRNA expression of several genes using quantitative polymerase chain reaction (qPCR). In both human PBMC-derived T cells and mouse spleen-derived T cells, isobutyric acid significantly decreased FOXP3 mRNA expression (Fig. 3), consistent with the decrease in FOXP3-positive T cells in the co-culture setting (Fig. 2a). INFG mRNA expression was significantly increased in a concentration-dependent manner in both human and mouse T cells (Fig. 3). PDCD1 mRNA expression was significantly upregulated by isobutyric acid in human T cells (Fig. 3a). Similarly, a concentration-dependent upregulation of PDCD1 was observed in mouse T cells, although the difference was not significant. ICOS mRNA was upregulated only in human T cells at higher concentrations of isobutyric acid, although the differences were not statistically significant (Fig. 3a). ICOS expression in mouse T cells was slightly increased by 10 mM isobutyric acid treatment (Fig. 3b). Further, qPCR results obtained with mono-cultured T cells were largely consistent with those of flow cytometric analysis in the co-culture setting, highlighting the direct effect of isobutyric acid on T cells.

### In vivo anti-cancer effect of isobutyric acid

**Figure 4:**
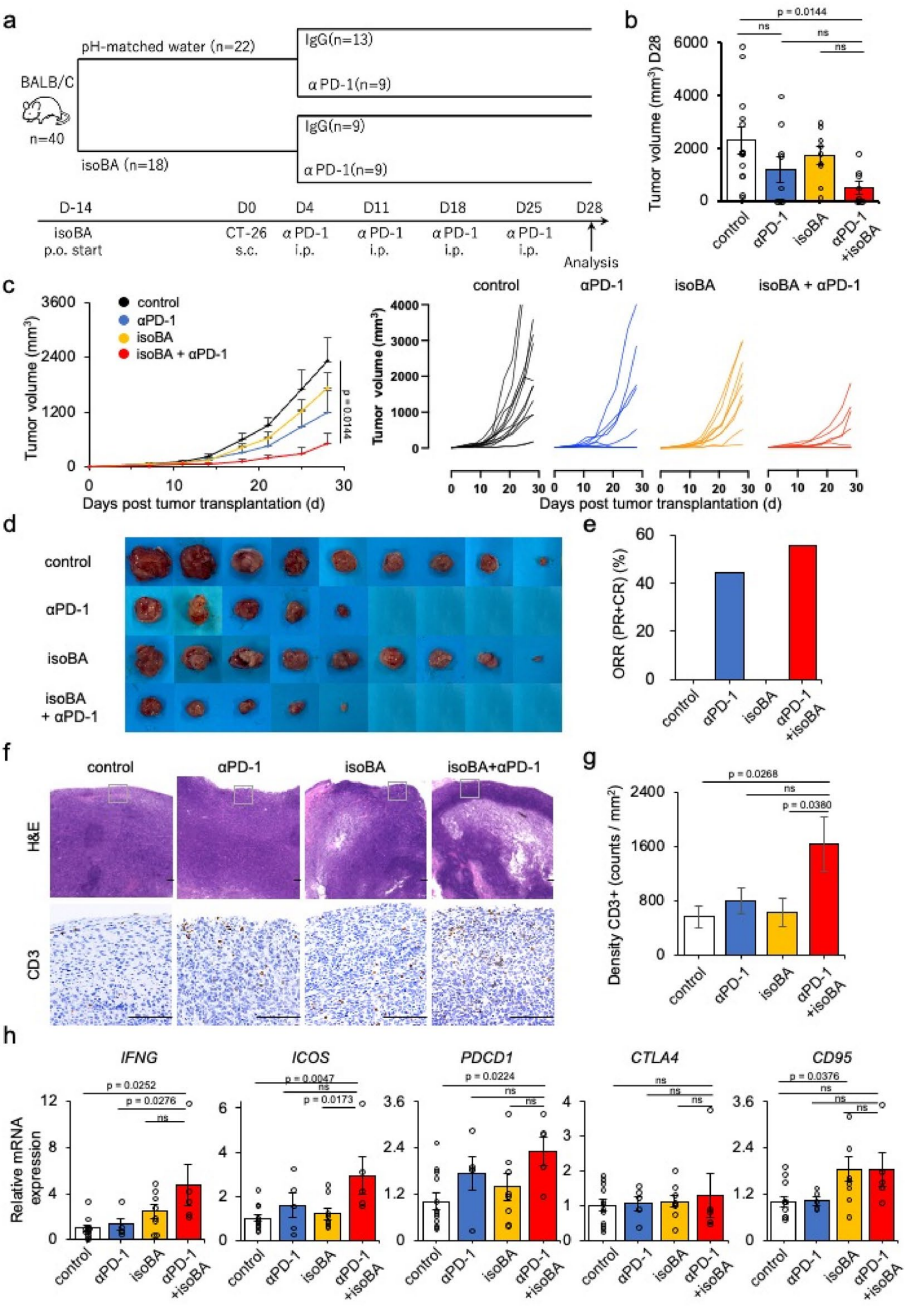
Anti-cancer effect of isobutyric acid in mice treated with an anti-PD-1 antibody. (**a**) BALB/C mice with CT26 cancers (*n* = 40) were pre-treated with orally administered 100 mM isobutyric acid or pH-matched water and intraperitoneally injected with 150 μg/150 μL of anti-PD-1 antibody or IgG on days 4, 11, 18, and 25 after cancer cell inoculation. (**b**) Tumour volume was analysed on day 28 after inoculation of tumour cells. (**c**) CT26 tumour growth curves. (**d**) Pictures of removed tumours. Pictures without a tumour on it represent a tumour that has disappeared. (**e**) Objective response rate (ORR). (**f**,**g**) Haematoxylin and eosin (H&E) staining, and CD3 immunostaining of excised cancer tissues. Scale bar 100 μm. (**h**) *IFNG*, *ICOS*, *PDCD1, CTLA-4, CD69, and CD95* levels in explanted cancer tissues. Data were obtained from two independent experiments. Statistical analysis was performed using ANOVA with Dunnett’s multiple comparison test. *ns* not significant. Error bars represent S.E.M.

Because isobutyric acid upregulated PD-1 expression in T cells, we hypothesised that combination of anti-PD-1 antibody and isobutyric acid had synergistic anti-tumour effect in an in vivo mouse model (Fig. 4a). Herein, 8–12-week-old male BALB/C mice were pre-treated with orally administered 100 mM isobutyric acid or pH-matched-water, and 2 × 10^5^ cells/50 μL CT26 colon cancer cells were injected subcutaneously 2 weeks later. We decided the concentration of isobutyric acid to feed mice according to a previous study^17^ in which mice received drinking water containing 100 mM butyric acid. Although mice treated with isobutyric acid drank slightly less water than those treated with pH-matched water (5.7 mL per mouse a day in the control group vs 4.3 mL in the isobutyric acid group), the amount of water intake of each group was within the normal range^21^. Anti-PD-1 antibody or IgG (150 μg/150 μL) was injected intraperitoneally on days 4, 11, 18, and 25 after cancer cell inoculation, and the tumours were removed 28 days later. Although not significant, both anti-PD-1 monotherapy and isobutyric acid monotherapy reduced tumour volume (Fig. 4b,c). Interestingly, combination therapy with anti-PD-1 antibody and isobutyric acid significantly suppressed tumour growth (p = 0.0144) (Fig. 4b–e). As reported previously [Wong et al., Nature comm (2023)14:5983], anti-PD-1 therapy showed dichotomous responses dividing mice into responder and non-responder groups (Fig. 4c,d). Overall response rate (complete response plus partial response rates) of combination therapy with anti-PD-1 antibody and isobutyric acid was slightly higher than that of anti-PD-1 monotherapy (Fig. 4e). Individual tumors growth of non-responder mice treated with isobutyric acid plus anti-PD-1 antibody were substantially smaller than that with anti-PD-1 antibody alone (Fig. 4c). These results indicate that isobutyric acid enhances anti-tumour effect of anti-PD-1 antibody. Immunostaining of the cancer tissues further revealed that the number of tumour-infiltrating CD3 + T cells significantly increased with anti-PD-1 antibody and isobutyric acid combo therapy (Fig. 4f,g). In addition, the gene expression of IFNG, ICOS, and PDCD1 significantly increased within tumour tissue upon the combination therapy (Fig. 4h). An activation marker CD95 also increased by isobutyric acid monotherapy. There were no significant differences in mRNA expression of CTLA-4 (Fig. 4h).

**Figure 5:**
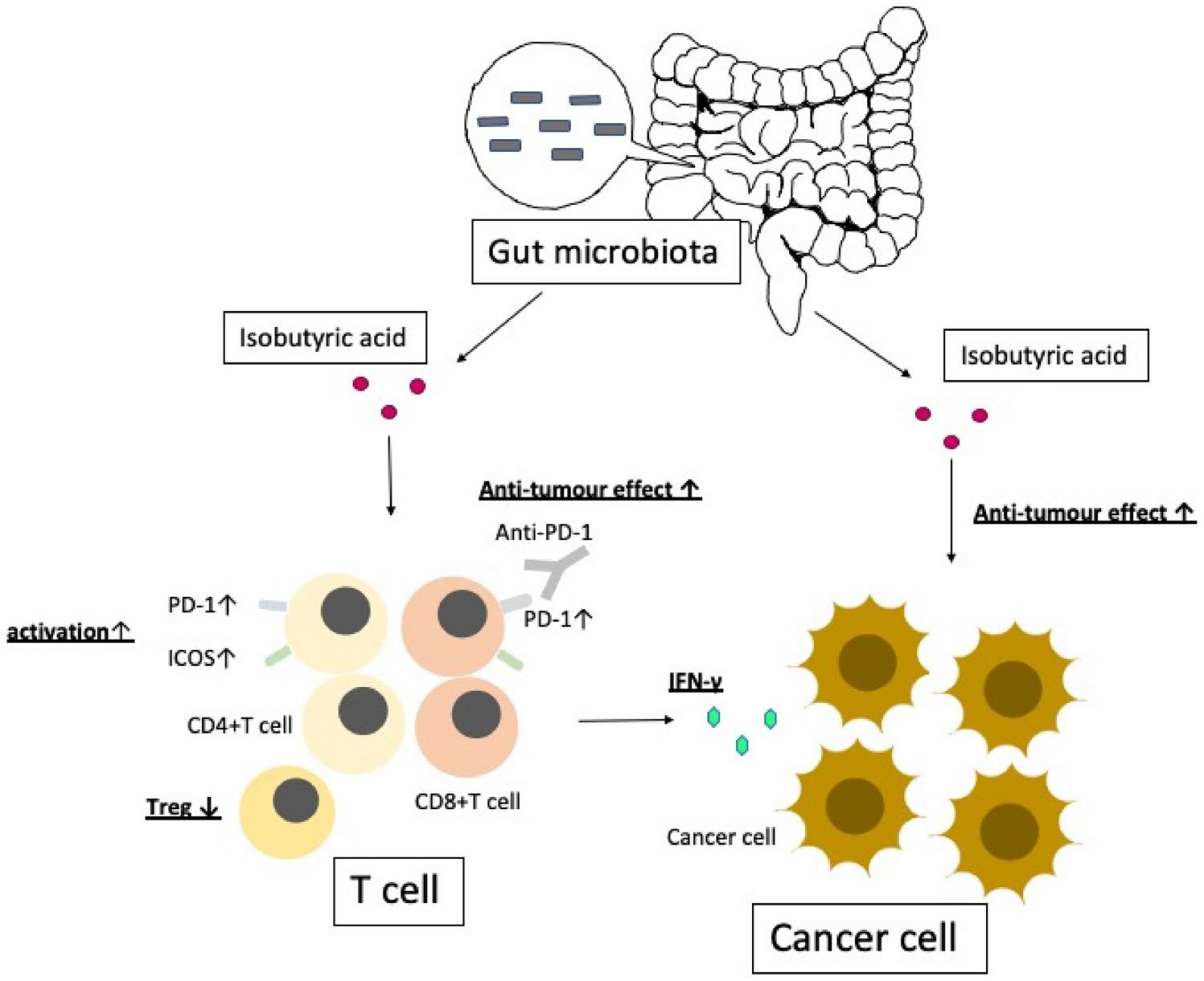
Overview of the effects of isobutyric acid on cancer and T cells in the tumour microenvironment. Isobutyric acid exerts a direct anti-tumour effect on cancer cells. Isobutyric acid suppresses Tregs, activates effector T cells, and increases the number of T cells expressing PD-1. Isobutyric acid enhances the efficacy of anti-PD-1 immunotherapy through its direct action on cancer cells and activation of T cells.

We also examined whether immunosuppressive cytokines such as IL-10 or TGF beta had any roles in enhancement of anti-tumour effect by combination of anti-PD-1 antibody plus isobutyric acid. Expression of these cytokines was not affected by anti-PD-1 antibody and/or isobutyric acid (Fig. S6), suggesting that these cytokines were not mechanistically involved. Furthermore, isobutyric acid did not increase the surface expression of PD-L1 or MHC class I molecules on tumor cells in terms of percentage and mean fluorescent intensity (Fig. S7), suggesting that augmenting effect of isobutyric acid was not attributed by expression of immune checkpoint ligands or antigen presentation on tumor cells. These results suggested that isobutyric acid can effectively enhance the efficacy of anti-PD-1 immunotherapy through direct anti-cancer effects on cancer cells and through the activation of T cells (Fig. 5).

## Discussion

Isobutyric acid, a branched SCFA, is produced by intestinal bacteria via fermentation of indigestible proteins, unlike straight SCFAs, which are produced from indigestible carbohydrates. Its concentration is much lower than those of other SCFAs, and its function in anti-tumour immunity is largely unknown. In this study, we investigated the effects of isobutyric acid on anti-tumour immunity in vitro and in vivo.

In vitro, isobutyric acid showed growth inhibitory effects on cancer cells rather than T cells in humans and mice. For T cells, isobutyric acid increased the expression of PD-1, an immune checkpoint molecule, and interferon gamma, a cytokine that enhances anti-tumour immunity. It also decreased the expression of FoxP3, a master transcription factor for regulatory T cells, as well as the percentage of regulatory T cells. All these indicate that isobutyric acid enhances anti-tumour immunity. In general, SCFAs are known to act via several mechanisms, including exhibiting agonistic action on GPCRs, inhibition of histone deacetylase (HDAC), and regulation of energy metabolism. Le Paul et al. reported that isobutyric acid had agonistic effects on GPR41 and GPR43 in a heterologous expression system using HEK293T cells^22^. The pEC50 values of isobutyric acid for these receptors are 3.2–3.8 and 4.3–4.5, respectively. This is in accordance with our results showing the action of isobutyric acid at the mM level. Thus, the effects of isobutyric acid on T cells observed in vitro herein could be partially attributed to its action on these GPCRs. Luu et al. examined SCFAs for their inhibitory effect on HDAC, showing that isobutyric acid had little inhibitory effect on HDAC compared to other SCFAs^17^. Therefore, it is unlikely that our results are due to HDAC inhibition. In addition, straight SCFAs, mainly acetic acid, propionic acid, and butyric acid, are known to promote T cell effector function through the mTOR-mediated regulation of glycolysis pathway^17^. However, such metabolic action has not been reported for isobutyric acid so far. Thus, further investigation is needed in this regard. Recently, Zhu et al. reported that isobutyric acid, like butyric acid, has the potential to modify lysine residues on histones^23^. They showed that histone acetyltransferases, such as HAT1 and p300, had lysine isobutyryltransferase activity in vitro and that cellular histone isobutyrylation level was regulated by p300. This is another possible mechanism by which isobutyric acid exerted its effects on both T cells and cancer cells.

Most notably, in this study, isobutyric acid showed synergistic anti-tumour effects with anti-PD-1 antibody. In a subcutaneous tumour model, isobutyric acid enhanced the anti-tumour effect of anti-PD-1 antibody. The expression of PD-1 and IFNG in the tumour tissue and the number of T cells infiltrating the tumour were also increased by the combination of isobutyric acid and anti-PD-1 antibody. These results partially replicate the in vitro results and strongly suggest that isobutyric acid enhances anti-tumour immunity in vivo. In the tumour microenvironment, however, in addition to cancer cells and T cells, various other components influence anti-tumour immunity, including macrophages, tumour-associated fibroblasts, and angiogenesis. Therefore, examining the effects of isobutyric acid on these cells will help to elucidate its anti-tumour mechanism.

This study has several implications for cancer immunotherapy. Nomura et al. examined the association of faecal and plasma SCFAs with response to anti-PD-1 antibody therapy in solid tumours and found that faecal isobutyric acid concentrations in responders were higher than those in non-responders^24^. In addition, faecal isobutyric acid concentrations were significantly high in colorectal cancer patients who responded to preoperative radiation and chemotherapy^25^. Furthermore, a correlation between response to capecitabine and faecal isobutyric acid concentration has been reported in patients with metastatic or unresectable colorectal cancer^26^. These results indicate that isobutyric acid may be a biomarker to predict efficacy in various cancer therapies. They also imply that isobutyric acid could be an adjuvant to anticancer chemotherapy and radiation therapy. This study also implies the usefulness of prebiotics inducing isobutyric acid production in cancer therapy. Aguirre et al. used an in vitro gut model to show that a high-protein diet increases faecal isobutyric acid concentrations^27^. A dietary intervention study also indicated that high-protein and low-fibre diet increased faecal concentration of branched SCFAs, such as isobutyric acid^28^. Isobutyric acid is produced from the branched-chain amino acid valine^29^. Thus, intake of indigestible proteins or valine may lead to increased isobutyric acid production by intestinal bacteria, potentiating the effects of ICIs.

In conclusion, our study revealed that isobutyric acid exhibited a synergistic anti-tumour effect with anti-PD-1 antibody. This discovery may lead to the development of adjuvant anti-cancer therapies to improve the response to ICIs.

## Methods

### Cell lines and culture conditions

CT-26 mouse colon cancer cells and T3M-1 Clone2 human oral cancer cells (American Type Culture Collection, Manassas, VA, USA) were cultured in RPMI-1640 medium (FUJIFILM, Tokyo, Japan) and Ham’s F-10 Nutrient Mix medium (Thermo Fisher Scientific, Waltham, MA, USA), respectively, supplemented with 10% foetal bovine serum and 1 × penicillin/streptomycin (both from Thermo Fisher Scientific). SW480 and MC38 (American Type Culture Collection, Manassas, VA, USA) were both grown in DMEM with high glucose concentration supplemented with 10% foetal bovine serum and 1 × penicillin/streptomycin. All cell lines were maintained at 37 °C in 5% CO_2_ atmosphere.

### Mouse T-cell activation/proliferation

T lymphocytes were purified from the spleens of BALB/C mice by positive selection with CD90.2 microbeads (Miltenyi Biotec, Bergisch Gladbach, Germany). T cells were activated with plate-bound anti-mouse CD3ε (2 µg/mL, clone 145-2C11) and anti-mouse CD28 (1 µg/mL, clone 37.51) antibodies (BioLegend, San Diego, CA, USA) in the presence of 100 U/mL interleukin-2 (PROSPEC, East Brunswick NJ, USA). T cells were cultured in 25 cm^2^ cell culture flasks with RPMI-1640 supplemented with penicillin–streptomycin and 10% foetal bovine serum medium in a total volume of 10 mL and maintained at 37 °C under 5% CO_2_ atmosphere. The cells were diluted up to 1 × 10^6^ cells/mL by adding fresh culture medium supplemented with 20 U/mL interleukin-2 on day 3. On day 4, the T cells were used for co-culture with CT26 cells.

### Human T-cell activation/proliferation

PBMCs were isolated from the peripheral blood of healthy donors using Ficoll-Paque density gradient centrifugation. T cells were activated with plate-bound anti-human CD3 (2 µg/mL, clone OKT3) and anti-human CD28 (2 µg/mL, clone CD28.2) antibodies (BioLegend, San Diego, CA, USA) in the presence of 100 U/mL interleukin-2 (PROSPEC, East Brunswick NJ, USA). T cells were cultured in 25 cm^2^ cell culture flasks with iMediam for T medium (GC LYMPHOTEC, Tokyo, Japan) in a total volume of 10 mL and maintained at 37 °C in 5% CO_2_ atmosphere. The cells were diluted up to 5 × 10^5^ T cells/mL by adding fresh culture medium supplemented with 25 U/mL interleukin-2 on days 4, 7, and 10. On day 14, the T cells were used for co-culture with T3M-1 Clone2 cells. All protocols and experiments involving primary PBMCs were approved by the Institutional Review Board of the Showa University. Informed consent was obtained from all volunteers.

### Co-culture experiments

Activated T cells (5 × 10^5^ cells) were mixed with cancer cells (5 × 10^4^ cells) that were previously seeded (at least 2 h before) in 6-well plates in iMediam for T medium or RPMI-1640 supplemented as the above medium in a total volume of 2 mL. After another 2 h, SCFAs (acetic, propionic, butyric, isobutyric, valeric, isovaleric, or hexanoic acids) (Merck KGaA, Darmstadt, Germany) were added to the co-culture in increasing concentrations (0.1, 3, or 10 mM). In order to make a pH similar with that of 10 mM isobutyric acid treatment, hydrochloric acid was added at a final concentration of 10 mM. The co-culture was maintained at 37 °C in 5% CO_2_ atmosphere for 72 h. The absolute numbers of cancer cells and T cells were determined using flow cytometry using fluorescent counting beads (Flow-Count, Beckman coulter, Brea, USA). After collecting floating cells, adherent cells were trypsinised and collected into the same tube. Fluorescent counting beads were added to each sample tube before staining cells with FITC or BV650-conjugated anti-CD45 antibody. CD45-positive cells were defined as T cells and all others as cancer cells. The numbers of T cells and cancer cells were corrected by the number of fluorophore beads counted in each sample (Supplementary Fig. S2).

### In vivo experiments

BALB/C mice were obtained from CLEA Japan (Tokyo, Japan) and maintained at a constant temperature (23 ± 1 °C) with a 12 h light/dark cycle under specific pathogen-free conditions. The experimental protocols were approved by the Institutional Animal Care and Use Committee of the Showa University, and all experiments were conducted according to relevant guidelines and regulations. This study is a research report following the ARRIVE guidelines. Age-matched 8–12-week-old male mice were subcutaneously injected with 2 × 10^5^ CT26 cells in the right flank. Mice received intraperitoneal injections of 150 μg of anti-PD-1 (clone RMP1-14; Lebanon, NH, USA) per injection or its isotype control (IgG2b; Lebanon, NH, USA) on days 4, 11, 18, and 25. Mice received isobutyric acid (100 mM) in the drinking water or pH-match water (control group) for 2 weeks prior to tumour inoculation, and the water solution was changed weekly. Tumour size was measured using a calliper twice a week until it reached the size that should be sacrificed, and tumour volume was determined as length × width^2^ × 0.5.

### Flow cytometry

After 72 h of co-culture, T and cancer cells were collected and pre-incubated with purified anti-mouse CD16/32 (TONBO, San Diego, USA) or Fc block (BD Biosciences, Franklin Lakes, NJ, USA) for 10 min at 20 °C before staining. The Transcription Factor Buffer Set (BD Biosciences, Franklin Lakes, NJ, USA) was used for intracellular staining. Antibodies used in flow cytometry experiments in this study are listed in Supplementary Table S1. When measuring the number of cells using polystyrene fluorospheres beads, a pre-determined number of beads was added to each pre-stained sample collected from the culture medium, and the number of cells after collection was calculated from the number of beads obtained using flow cytometry. CD45-positive cells were defined as T cells and all others as cancer cells. Flow cytometry was performed using a BD LSFortessa coupled with FACSDiva v6.1.3 and data analysed using FlowJo v10.7.1 software (all from BD Biosciences).

### Tumour immunostaining

All samples were fixed in 10% formalin (WAKO, Osaka, Japan) for 24 h, and serial sections of 4-μm thickness were affixed to and dried on silane-coated slide glasses. After deparaffinisation, antigens were retrieved by pre-treating the sections with citrate buffer (pH 6.0; AS ONE, Osaka, Japan) for 20 min at 121 °C in an autoclave. Sections of cancer tissues were prepared for staining by treating with 0.3% H_2_O_2_ for 10 min to remove endogenous peroxidase, followed by blocking with non-specific blocking reagent (DAKO, Tokyo, Japan) for 5 min. Sections were then incubated with the primary antibody, anti-CD3 antibody (1:100; Abcam, Cambridge, UK) for 1 h, followed by Envision/HRP-labelled secondary antibody (DAKO) for 30 min. The colour was developed using the DAKO DAB Liquid System. CD3^+^ cells were counted using cancer tissue slides from five mice from each treatment group, per six high-power fields, in a blinded manner. CD3^+^ cells are expressed as mean ± SEM of CD3^+^/mm^2^. Images were acquired using a BZ-X700 microscope (Keyence, Osaka, Japan) operated on a BZ-X800 Viewer.

### RNA isolation and quantitative analysis

Total RNA was isolated from cancer tissues using an RNAeasy mini kit in conjunction with a QIAshredder (Qiagen, Hilden, Germany), according to the manufacturer’s instructions. The concentration and purity of the isolated RNA was confirmed using a NanoDrop Lite Spectrophotometer (Thermo Fisher Scientific). RNA was then reverse-transcribed using the High-Capacity cDNA Reverse Transcription Kit (Thermo Fisher Scientific) according to the manufacturer’s instructions. Target cDNA was amplified by PCR using QuantStudio3 with TaqMan Fast Advanced Master Mix (Thermo Fisher Scientific), according to the manufacturer’s protocol. The primers were purchased from Integrated DNA Technologies (Coralville, IA, USA). Relative gene expression level was calculated using the comparative Ct method and expressed as fold changes relative to the control. HPRT and ACTB were used as internal control genes for human and mouse gene expression, respectively.

### Statistical analysis

Data were analysed using ANOVA with Dunnett’s multiple-comparison test or unpaired two-tailed Student’s *t*-test. Values are presented as mean ± S.E.M. *P*-values < 0.05 were considered to indicate statistical significance.

### Supplementary Information

Supplementary Information.

## Data Availability

The data that support the findings of this study are available from the corresponding author upon reasonable request.
