# Supplementary material for: Isobutyric acid enhances the anti-tumour effect of anti-PD-1 antibody
Source: Sci Rep. 2024 May 17;14:11325. doi: 10.1038/s41598-024-59677-1 (PMC11101641; doi:10.1038/s41598-024-59677-1)
Supplement: Supplementary file 1 — Supplementary Information. [file 41598_2024_59677_MOESM1_ESM.pdf]

Supplementary Information

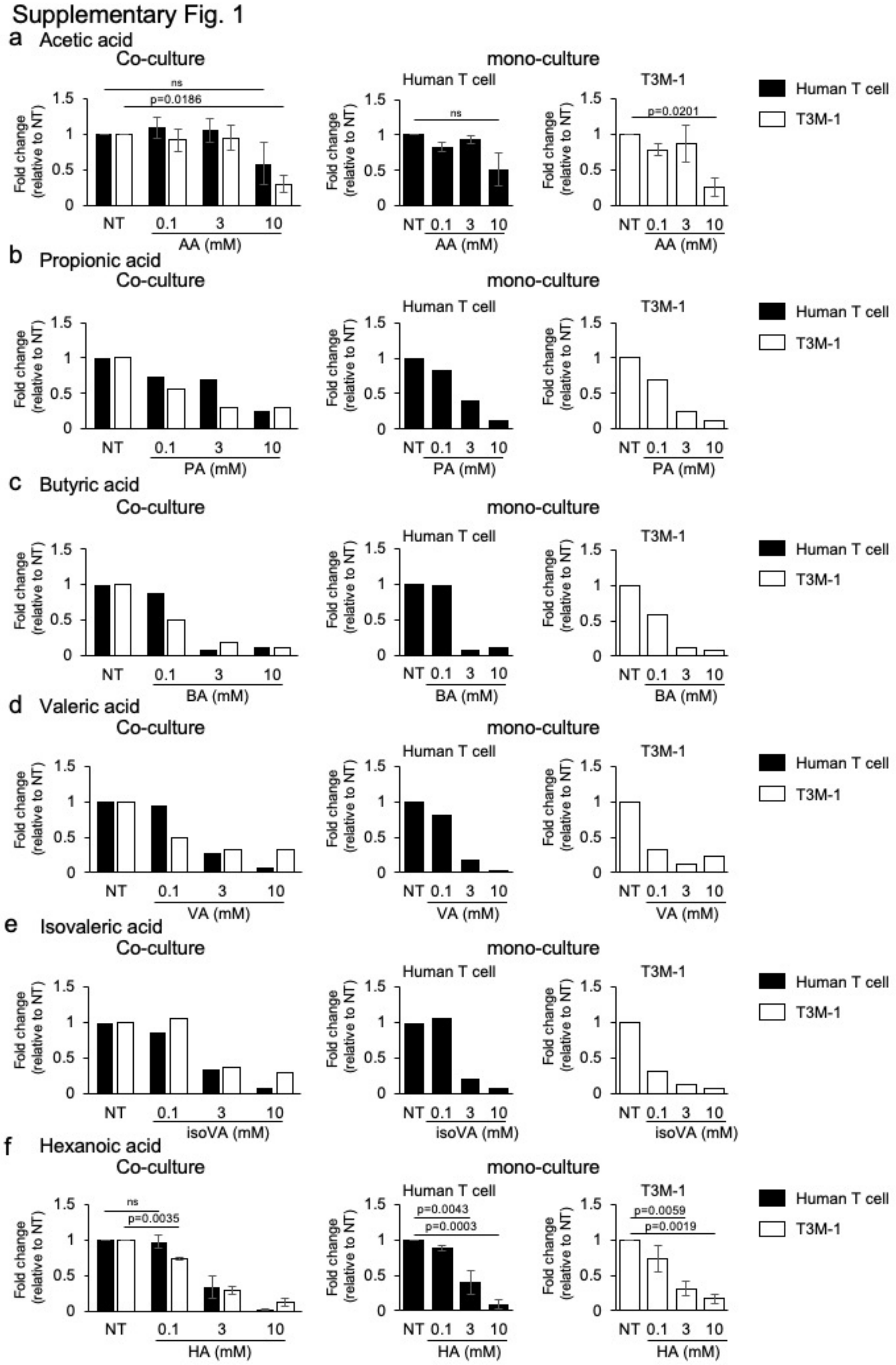

Figure S1. Effects of various short chain fatty acids on human T cells and T3M-1 cancer cells. Human T cells (5 × 10^5 cells) and T3M-1 Clone2 oral cancer cells (5 × 10^4 cells) were co- or mono-cultured for 72 h with **a,** acetic acid; **b**, propionic acid; **c**, butyric acid; **d**, valeric acid; **e**,

isovaleric; and **f**, hexanoic acid (at the indicated concentrations. The number of cancer and T cells was analysed using flow cytometry using fluorescent counting beads. CD45-positive cells were defined as T cells and all others as cancer cells. The graphs in the left, centre, and right represent cancer/T cells co-culture, T cell mono-culture, and cancer cell mono-culture, respectively. Experiments using acetic acid and hexanoic acid were repeated three times and others were conducted only once. Error bars represent S.E.M. NT, no treatment; AA, acetic acid; PA, propionic acid; BA, butyric acid; VA, valeric acid; isoVA, isovaleric acid; HA, hexanoic acid; ns, not significant.

Supplementary Fig. 2

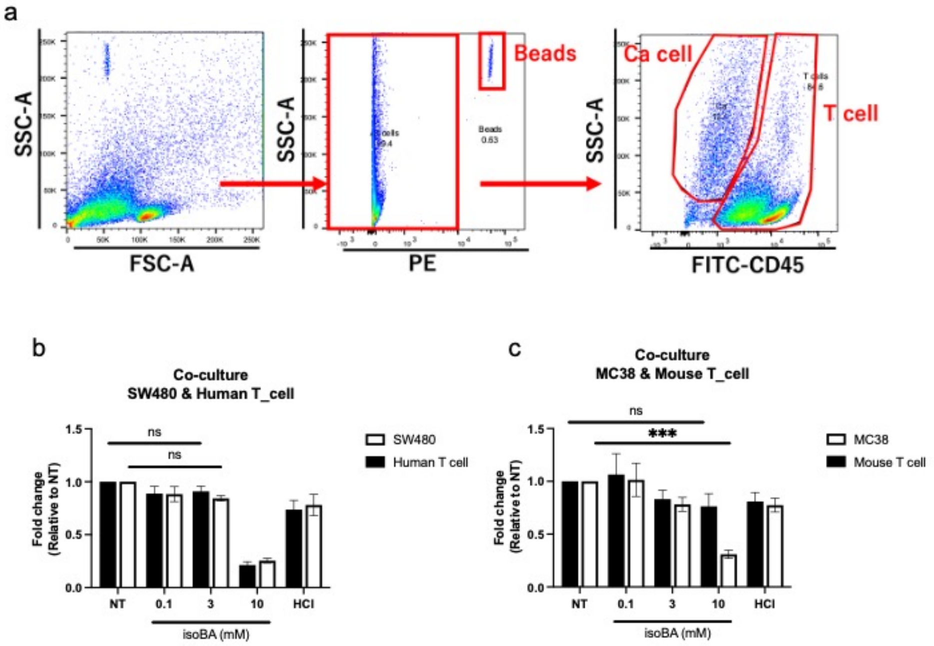

Figure S2. Effect of isobutyric acid on human and mouse colon cancer cells and T cells in co-culture setting. a, Gating strategy to count T cells and cancer cells separately using flow cytometry. Fluorescent counting beads were included in the sample before staining with FITC-CD45. Beads were gated on the PE-channel, and the remaining population was further separated into CD45-positive T cells and negative cancer (Ca) cells. **b,** Human T cells (5×10^5 cells) and SW480 colon cancer cells (5×10^4 cells) were co-cultured for 72 h with isobutyric acid at the indicated concentrations. **c**, Mouse T cells and MC38 colon cancer cells were co-cultured for 72 h with isobutyric acid at the indicated concentrations. To examine the effect of acidic conditions, hydrochloric acid was added at a final concentration of 10 mM to achieve a pH similar to that of isobutyric acid. Data are mean of three independent experiments. Error bars represent S.E.M. NT, no treatment; HCl, hydrochloric acid; isoBA, isobutyric acid; ns, not significant; ***$p<0.001$ vs NT.

Supplementary Figure 3

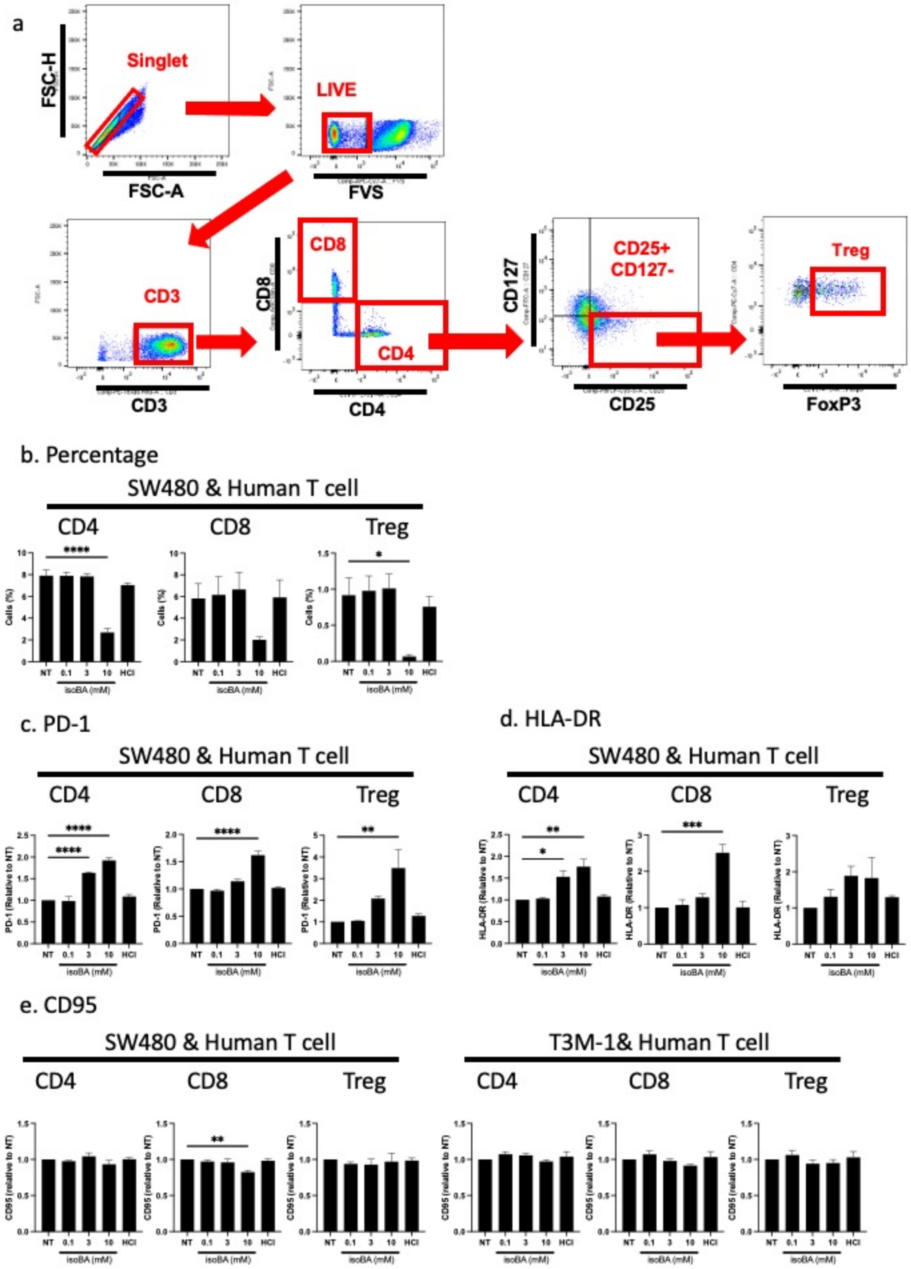

Figure S3. Characterisation of human T cell populations co-cultured with cancer cell lines upon treatment with isobutyric acid. a, Gating strategy to define human CD4+, CD8+ and Treg (CD4+ CD25+ CD127- FoxP3+) cells. **b,** Percentages of CD4+, CD8+, and Treg cells present in co-culture of SW480 colon cancer and T cells with various concentrations of

isobutyric acid. **c-e**, Expression of PD-1 (c), HLA-DR (d) and CD95 (e) in CD4+ T cells, CD8+ T cells, and Treg cells in co-culture setting. To examine the effect of acidic condition, hydrochloric acid was added at 10 mM to induce a low pH as that by isobutyric acid. Data are mean of three independent experiments. Error bars represent S.E.M. NT, no treatment; HCl, hydrochloric acid; isoBA, isobutyric acid; *$p<0.05$, **$p<0.01$, ***$p<0.001$, ****$p<0.0001$ vs NT.

Supplementary Figure 4

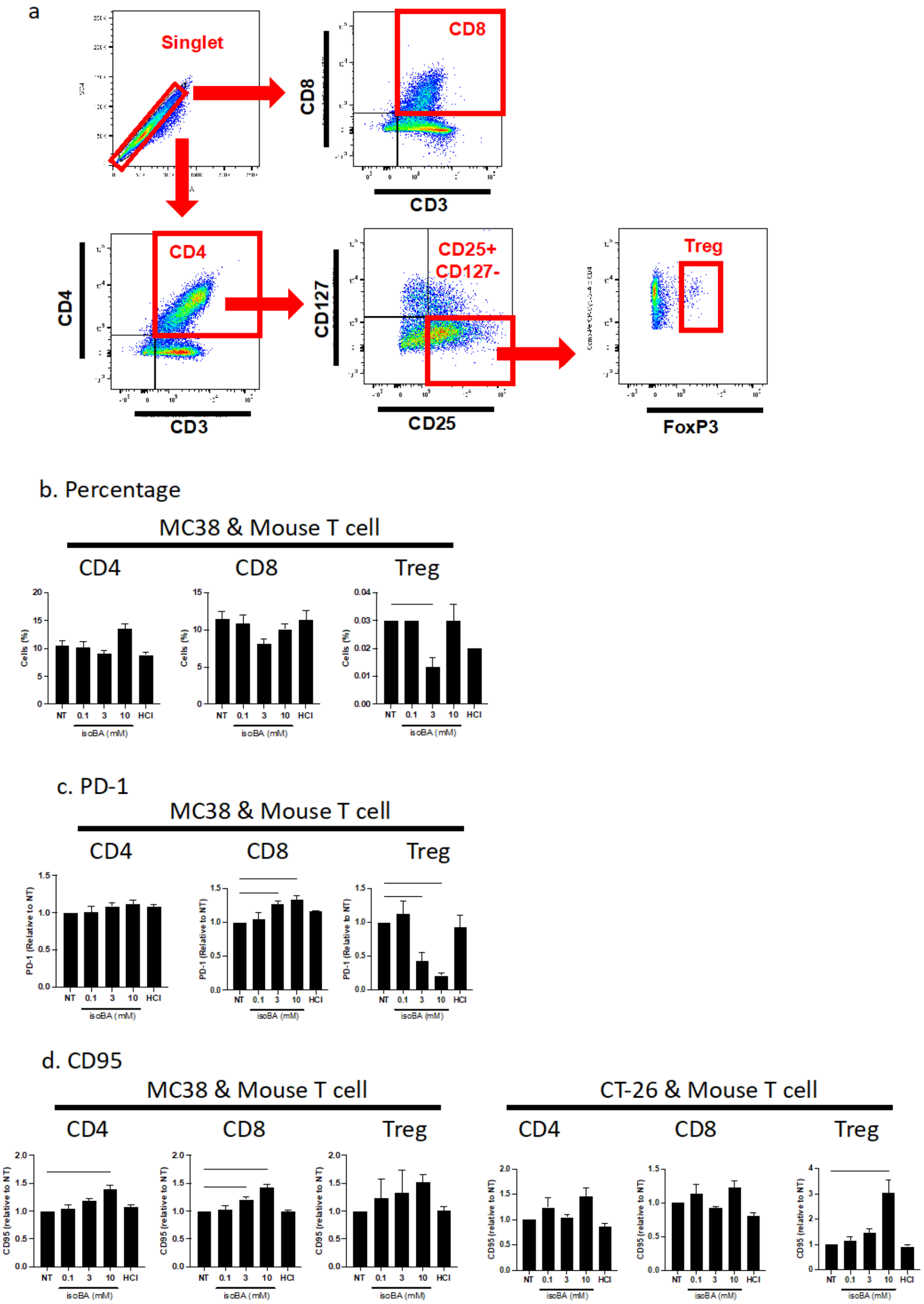

Figure S4. Characterisation of mouse T cell populations co-cultured with cancer cell lines upon treatment with isobutyric acid. a, Gating strategy to define mouse CD4+, CD8+ and Treg (CD4+ CD25+ CD127- FoxP3+) cells. **b,** Percentages of CD4+, CD8+, and Treg cells

present in co-culture of MC38 colon cancer and T cells with various concentrations of isobutyric acid. **c,d**, Expression of PD-1 (c) and CD95 (d) in CD4+ T cells, CD8+ T cells, and Treg cells in co-culture setting. To examine the effect of acidic condition, hydrochloric acid was added at 10 mM to induce a low pH as that by isobutyric acid. Data are mean of three independent experiments. Error bars represent S.E.M. NT, no treatment; HCl, hydrochloric acid; isoBA, isobutyric acid; *p<0.05, **p<0.01, ***p<0.001 vs NT.

Supplementary Figure 5

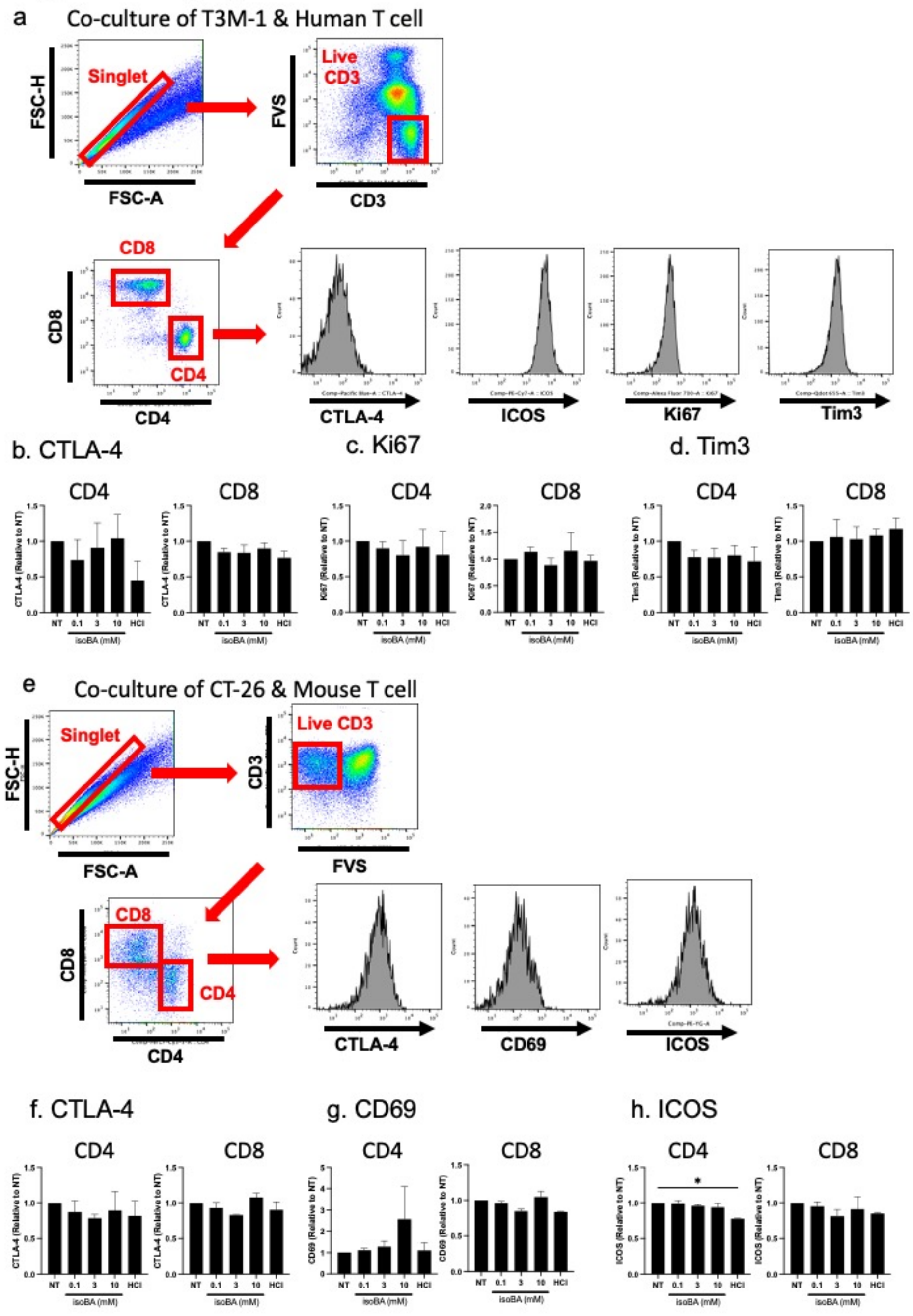

Figure S5. Effect of isobutyric acid on the phenotype of T cells in co-culture setting. a, Gating strategy to define human CD4+ and CD8+ T cells with representative histograms used to evaluate the expression of various markers. **b-d,** Expression of CTLA-4 (**b**), Ki67 (**c**), and Tim3 (**d**) in human CD4+ and CD8+ Tcells. **e**, Representative plots to gate mouse CD4+ and CD8+ T

cells with histograms of CTLA-4, CD69, and ICOS expression. **f-h,** Expression of CTLA-4 (**f**), CD96 (**g**), and ICOS (**h**) in mouse CD4+ and CD8+ Tcells. Data are mean of three independent experiments. Error bars represent S.E.M. NT, no treatment; HCl, hydrochloric acid at 10 mM; isoBA, isobutyric acid; *p<0.05 vs NT.

Supplementary Figure 6

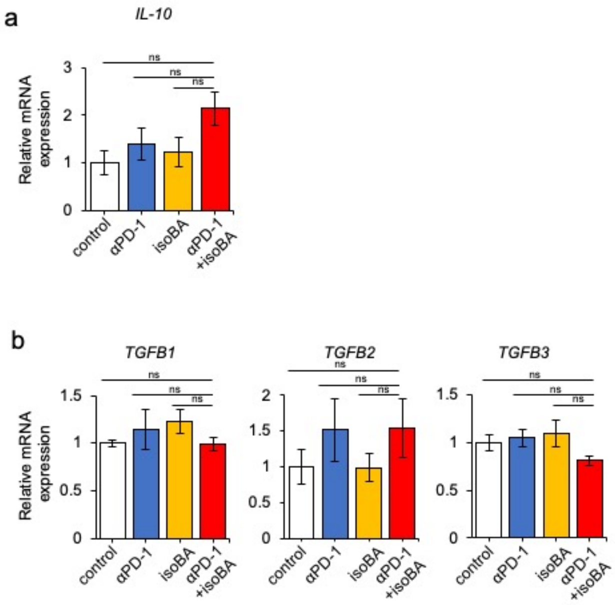

Figure S6. Expression of immunosuppressive cytokines in tumour tissue from CT-26 subcutaneous tumour model. Tumour tissues were isolated from mice with subcutaneous CT-26 tumour treated with isobutyric acid (isoBA) and/or anti-PD-1 antibody (αPD-1). Messenger RNA expression of IL-10 (**a**) and TGFbeta1-3 (**b**) was evaluated by quantitative RT-PCR. Data are mean of nine to thirteen independent experiments. Error bars represent S.E.M. ns, not significant.

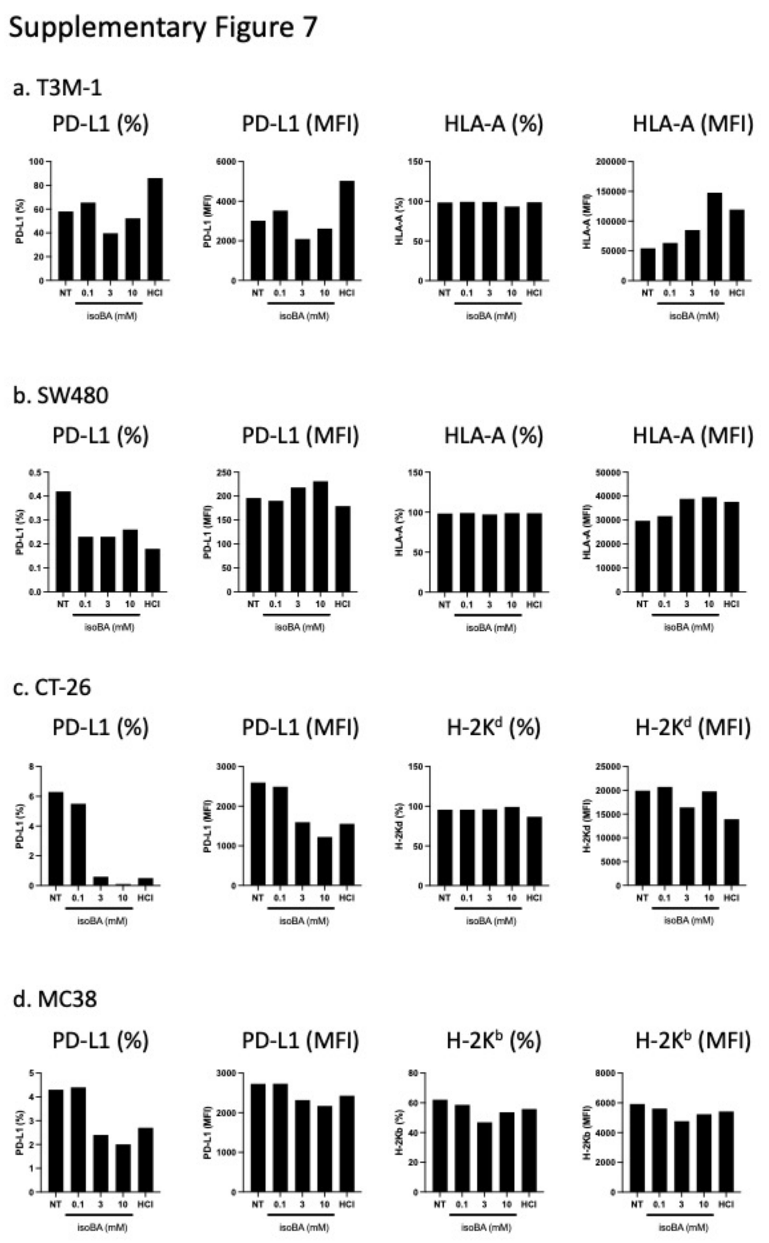

Figure S7. Effect of isobutyric acid on expression of PD-L1 and class I major histocompatibility (MHC-I) molecules on human and mouse tumour cell lines.

Tumour cell lines were treated with different concentrations of isobutyric acid and surface expression of PD-L1 and MHC-I (HLA-A for human cells; H-2K^d and H-2K^b for CT-26 and MC38, respectively) were analysed by flow cytometry. Percentage (%) and mean fluorescent intensity (MFI) are shown for each molecule in T3M-1 (**a**), SW480 (**b**), CT-26 (**c**) and MC38

(**d**). HCl, hydrochloric acid at 10 mM; isoBA, isobutyric acid. Shown are representative data from two independent experiments with similar results.

Supplementary Table 1. Antibodies used in flow cytometry.

Panel 1 (Human T cell for Figures 2a-c and S3)					
species	target	clone	fluorophore	Supplier	catalog no
Human	CD3	HIT3a	PE-Dazzle	BioLegend	300336
Human	CD4	RPA-T4	PE-Cy7	BioLegend	300512
Human	CD8a	RPA-T8	BV510	BioLegend	301048
Human	CD25	M-A251	PerCP-Cy5.5	BioLegend	356112
Human	CD95	DX2	Alexa Fluor 700	BioLegend	305647
Human	CD127	A019D5	FITC	BioLegend	351312
Human	PD-1	EH12.2H7	PE	BioLegend	329906
Human	FoxP3	206D	Alexa Fluor 647	BioLegend	320114
Human	HLA-DR	L243	Pacific Blue	BioLegend	307623

Panel 2 (Mouse T cell for Figures 2a, b and S4)					
species	target	clone	fluorophore	Supplier	catalog no
Mouse	CD3	17A2	Alexa Fluor 647	BioLegend	100216
Mouse	CD4	RM4-5	PerCP-Cy5.5	BioLegend	100540
Mouse	CD8a	53-6.7	BV510	BioLegend	100752
Mouse	CD25	PC61	FITC	BioLegend	102006
Mouse	CD95	SA367H8	APC	BioLegend	152604
Mouse	CD127	A7R34	APC-Cy7	BioLegend	135039
Mouse	PD-1	29F.1A12	PE-cy7	BioLegend	135216
Mouse	FoxP3	R16-715	PE-CF594	BD Biosciences	567373

Panel 3 (Human T cell for Figures 2d and S5a-d)					
species	target	clone	fluorophore	Supplier	catalog no
Human	CD3	HIT3a	PE/Dazzle	BioLegend	300336
Human	CD4	RPA-T4	PerCP-Cy5.5	BioLegend	300530
Human	CD8a	RPA-T8	BV510	BioLegend	301048

Human	CD45	HI30	FITC	BioLegend	304006
Human	ICOS	C398.4A	PE-Cy7	BioLegend	313519
Human	PD-1	EH12.2H7	PE	BioLegend	329906
Human	FoxP3	206D	Alexa Fluor 647	BioLegend	320114
Human	CTLA-4	BNI3	BV421	BioLegend	369606
Human	Ki67	Ki-67	Alexa Fluor 700	BioLegend	350530
Human	Tim3	F38-2E2	BV650	BioLegend	345028

Panel 4 (Mouse T cell for Figures S5e-h)					
species	target	clone	fluorophore	Supplier	catalog no
Mouse	CD3	17A2	Alexa Fluor 700	BioLegend	100216
Mouse	CD4	GK1.5	PE-Cy5	eBioscience	15-0041-82
Mouse	CD8a	53-6.7	BV510	BioLegend	100752
Mouse	CD45	30-F11	BV650	BioLegend	103151
Mouse	CD69	H1.2F3	PE/Dazzle	BioLegend	104536
Mouse	FoxP3	MF-14	BV421	BioLegend	126419
Mouse	PD-1	29F.1A12	PE-Cy7	BioLegend	135216
Mouse	ICOS	15F9	PE	BioLegend	107706
Mouse	CTLA-4	UC10-4B9	APC	BioLegend	106310

Antibodies used in Figure S7					
species	target	clone	fluorophore	Supplier	catalog no
Human	PD-L1	MIH1	PE-Cy7	BD Biosciences	558017
Human	HLA-A	1082C5	PE	BD Biosciences	567739
Mouse	PD-L1	10F.9G2	BV421	BioLegend	124315
Mouse	H-2Kb	AF6-88.5	FITC	BioLegend	116505
Mouse	H-2Kd	SF1-1.1	FITC	BioLegend	116605
